# Optogenetically Induced Seizure and the Longitudinal Hippocampal Network Dynamics

**DOI:** 10.1371/journal.pone.0060928

**Published:** 2013-04-10

**Authors:** Shin-ichiro Osawa, Masaki Iwasaki, Ryosuke Hosaka, Yoshiya Matsuzaka, Hiroshi Tomita, Toru Ishizuka, Eriko Sugano, Eiichi Okumura, Hiromu Yawo, Nobukazu Nakasato, Teiji Tominaga, Hajime Mushiake

**Affiliations:** 1 Department of Neurosurgery, Tohoku University Graduate School of Medicine, Sendai, Japan; 2 Department of Applied Mathematics, Fukuoka University, Fukuoka, Japan; 3 Department of Physiology, Tohoku University Graduate School of Medicine, Sendai, Japan; 4 Department of Chemistry and Bioengineering, Faculty of Engineering, Graduate School of Science and Engineering, Iwate University, Morioka, Japan; 5 Department of Developmental Biology and Neuroscience, Tohoku University Graduate School of Life Sciences, Sendai, Japan; 6 Department of Epileptology, Tohoku University Graduate School of Medicine, Sendai, Japan; 7 Center for Neuroscience, Tohoku University Graduate School of Medicine, Sendai, Japan; 8 Core Research of Evolutional Science & Technology (CREST), Japan Science and Technology Agency (JST), Tokyo, Japan; Consejo Superior de Investigaciones Cientificas - Instituto Cajal, Spain

## Abstract

Epileptic seizure is a paroxysmal and self-limited phenomenon characterized by abnormal hypersynchrony of a large population of neurons. However, our current understanding of seizure dynamics is still limited. Here we propose a novel *in vivo* model of seizure-like afterdischarges using optogenetics, and report on investigation of directional network dynamics during seizure along the septo-temporal (ST) axis of hippocampus. Repetitive pulse photostimulation was applied to the rodent hippocampus, in which channelrhodopsin-2 (ChR2) was expressed, under simultaneous recording of local field potentials (LFPs). Seizure-like afterdischarges were successfully induced after the stimulation in both *W-TChR2V4* transgenic (*ChR2V*-TG) rats and in wild type rats transfected with adeno-associated virus (AAV) vectors carrying ChR2. Pulse frequency at 10 and 20 Hz, and a 0.05 duty ratio were optimal for afterdischarge induction. Immunohistochemical c-Fos staining after a single induced afterdischarge confirmed neuronal activation of the entire hippocampus. LFPs were recorded during seizure-like afterdischarges with a multi-contact array electrode inserted along the ST axis of hippocampus. Granger causality analysis of the LFPs showed a bidirectional but asymmetric increase in signal flow along the ST direction. State space presentation of the causality and coherence revealed three discrete states of the seizure-like afterdischarge phenomenon: 1) resting state; 2) afterdischarge initiation with moderate coherence and dominant septal-to-temporal causality; and 3) afterdischarge termination with increased coherence and dominant temporal-to-septal causality. A novel *in vivo* model of seizure-like afterdischarge was developed using optogenetics, which was advantageous in its reproducibility and artifact-free electrophysiological observations. Our results provide additional evidence for the potential role of hippocampal septo-temporal interactions in seizure dynamics *in vivo*. Bidirectional networks work hierarchically along the ST hippocampus in the genesis and termination of epileptic seizures.

## Introduction

Epileptic seizure is a paroxysmal and self-limited phenomenon characterized by abnormal hypersynchrony of a large population of neurons [1]–[3]. Focal seizures, or seizures generated in a certain region of the brain, are usually initiated in the presence of pathological causes and propagated to distant normal cortex. The hippocampus and surrounding limbic structures are the sites most prone to focal epileptic seizures, especially those resistant to medications [4], [5]. However, our current understanding of seizure genesis (i.e., the mechanism that initiates epileptic seizures in complex neuronal networks) is still limited [6]–[8].

Electrical stimulation of the brain has long been used to induce epileptogenesis and epileptic seizures. Electrical kindling, the classic model of epileptogenesis, usually takes weeks to induce spontaneous seizures with less than 70% reproducibility [6], [9]. Trains of high-frequency electrical stimulations induce seizure-like patterns as “afterdischarges”. However, a pattern of the induced activity can be substantially different from spontaneous seizures in humans [7], [10]–[14]. Although this is a convenient model of epileptic seizures, the electrical stimulation generates large artifacts which interfere with neuronal activity recording in animal models, thus making it difficult to study the underlying physiological mechanism of the onset, propagation and cessation of seizure *in vivo*. Increasing the arsenal of seizure models is thus required to better pursue for the electrophysiological mechanisms underlying ictogenesis.

The above limitations of the conventional seizure models prompted us to apply optogenetic stimulation to the hippocampus to develop a new animal model of seizure. Optogenetics refers to the integration of optics and genetics to achieve fast excitation or inhibition of specific neurons in living tissues. Recent development of this technique has facilitated our understanding of disease mechanisms at functional circuit level [15], [16]. Optogenetics also enables therapeutic manipulation of neural function, which has been attempted in disease states, such as in Parkinson's disease [17]. However, few studies have been published so far in the field of epilepsy or epileptic seizures [16], [18], [19]. The advantages of optogenetic stimulation are threefold. First, like electrical stimulation, it allows temporally precise stimulation to neurons. Second, it is capable of manipulating the activity of specific groups of neurons. And finally, unlike electrical stimulation, the optogenetic stimulation does not generate stimulation artifact, thus making it possible to study electrophysiologically the spatio-temporal dynamics of neuronal activity during stimulation [20], [21].

We used optogenetic techniques to perturb the normal hippocampal network in an effort to induce seizure-like afterdischarges. The method was established as a novel *in vivo* model of epileptic seizures. Spatio-temporal dynamics of seizure genesis and the transition between the ictal and interictal states were investigated with simultaneous electrophysiological recordings.

## Materials and Methods

### Ethics statement

This study was carried out in strict accordance with the recommendation in the Regulations for Animal Experiments and Related Activities at Tohoku University. All animal experiments were approved by the institutional animal care and use committee at Tohoku University (approval number 57, year 2011). All surgery was performed under ketamine and xylazine anesthesia, and all efforts were made to minimize suffering.

### Subjects

*Thy1.2-ChR2V* transgenic rats (transgene-positive *W-TChR2V4*, male and female, 240–360 g) [22], [23] and wild-type rats (male Wistar, 200–300 g) were used in the following experiments.

### Repetitive photostimulation of the ChR2-expressing hippocampus and induction of seizure-like afterdischarges

**Figure 1:**
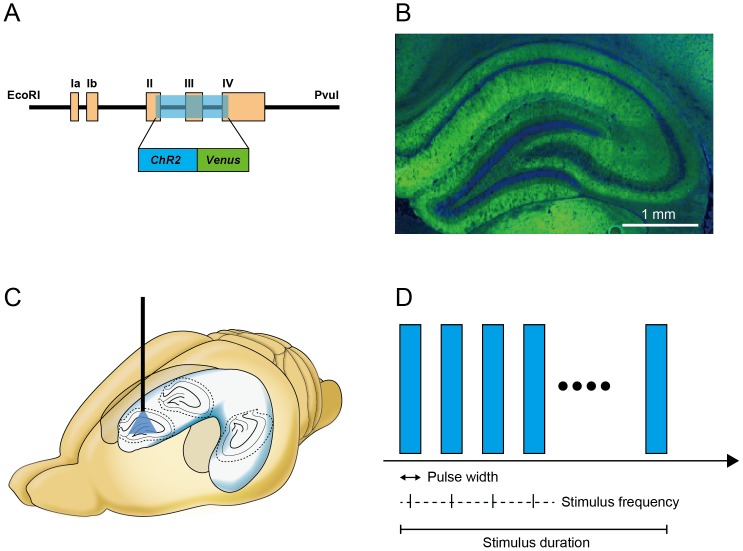
Optogenetic stimulation of *W-TChR2V4* transgenic (*ChR2V*-TG) rat hippocampus. (A) Schematic drawing of the DNA fragment inserted at the site of the modified mouse Thy-1.2 expression cassette. (B) *ChR2V*-TG rat hippocampal organization showed that ChR2V fluorescence (green) was dense in all layers except the cell layer marked with DAPI nuclear stain (blue). (C) Schematic drawing of photostimulation to the septal hippocampus of rat brain. (D) Repetitive pulse photostimulation is schematically presented. The duty ratio was calculated as the proportion of the pulse width to the inter-pulse interval.

First, we studied whether photic stimulation was able to induce seizure-like afterdischarges in the hippocampus of *W-TChR2V4* transgenic (*ChR2V*-TG) rats (Figure 1A), and if so, what stimulation parameters best induced seizure-like afterdischarges. The diffuse and strong expression of Venus protein was confirmed in the brain parenchyma of *ChR2V*-TG rats (Figure 1B). Repetitive pulse or continuous photostimuli were delivered to the CA3 – dentate gyrus (DG) portion of the septal hippocampus of anesthetized *ChR2V*-TG rats while LFP was simultaneously recorded using a hybrid optic fiber-electrode to observe neuronal activity during and after the stimulation (Figure 1C). The frequency (5, 10, 20, 40 Hz or continuous light) and the duty ratio (0.02, 0.05, 0.1, 0.3, 0.5, 0.7, or 0.9) of photostimulation were systemically varied to determine the optimal stimulation parameters for afterdischarge induction (Figure 1D). Optical intensity and stimulus duration were fixed at 19–22 mW at the tip of optic fiber and at 10 seconds, respectively. A total of 10 stimulation trials were performed for every combination of parameters (4 in frequency by 7 in duty ratio +1 for continuous light = 29 combinations of parameters, total 290 trials). The chance of afterdischarge induction was calculated for each stimulation parameter as a ratio of the number of induced afterdischarges in 10 trials of stimulation. A 5-minute minimum interval was made between trials, and no more than 30 trials were performed in a single animal. Parameters were randomized between trials and between animals. A total of ten *ChR2V*-TG rats were used.

Next, we examined whether afterdischarge induction was due to an innate susceptibility of *ChR2V*-TG animals to seizures. Repetitive photostimulation was applied to the hippocampus of wild-type Wistar rats transfected with the rAAV-ChR2-Venus vector (*n* = 3 rats). The AAV carrying a transgene encoding a ChR2V fusion protein driven by the hybrid cytomegalovirus enhancer/chicken beta-actin promoter was injected into the dentate hilus of the septal hippocampus in normal rats. The viral injection was targeted to 1 mm deeper than the site of stimulation, so that the tissue with ChR2 expression was exposed to photostimulus. Intense ChR2V expression in hippocampal neurons was confirmed 4–6 weeks later. Repetitive pulse photostimulation with combinations of parameters of frequency (5, 10, 20 Hz) and duty ratio (0.05, 0.1, 0.2) were delivered under simultaneous LFP recording using a hybrid electrode. Optical intensity and stimulus duration were fixed at 19–22 mW at the tip of optic fiber and at 20 seconds, respectively. Since the amount of ChR2 expression after viral transfection was expected smaller than in transgenic animals, longer stimulation duration was attempted.

Next, afterdischarge thresholds to electrical pulse stimulation were compared between *ChR2V*-TG rats and wild type Wistar rats (*n* = 5, each). A high-impedance (1–2 M ohm) monoplar electrode coupled with a bipolar stainless steel electrode was used (PlasticsOne, Roanoke, VA, USA). The afterdischarge threshold, a minimum current intensity that could induce afterdischarges, was measured in the septal hippocampal region. Bipolar electrical stimulation was applied with a constant current electric stimulator (SEN-3301, SS-102J; Nihon Kohden, Tokyo, Japan). Monophasic square pulses with a pulse duration of 1.0 ms were delivered at a frequency of 60 Hz for 1.0 s under the monitoring LFPs with the same settings as described above. The afterdischarge threshold was determined by delivering a series of graded intensities, which started at 50 µA and increased by 30% increments every 30 min until afterdischarge was triggered.

We also examined whether afterdischarge induction occurred in the hippocampus of wild-type Wistar rats and in the extra-hippocampal regions of *ChR2V*-TG rats. For wild-type rats (*n* = 3), repetitive pulse photostimulation was applied to the septal hippocampus. For *ChR2V*-TG rats (*n* = 4), repetitive pulse photostimulation was applied to the septal hippocampus, amygdala, anterior thalamus, and sensori-motor cortices under simultaneous LFP monitoring, similarly as performed in the hippocampus. Stimulation parameters were set at 10 Hz with a duty ratio of 0.05, the optimal parameters for afterdischarge induction in the hippocampus. Optical intensity and stimulus duration were fixed at 19–22 mW at the tip of optic fiber and at 10 seconds, respectively. Photostimulation was repeated 10 times in one location with minimum 5-minute interval, so that the chance of afterdischarge induction was calculated.

### Preparation of AAV vector carrying the ChR2 gene construct

The N-terminal fragment (residues 1–315) of ChR2 (GenBank accession no. AF461397) was fused to a fluorescent protein, Venus, in frame at the end of the ChR2-coding fragment (ChR2V). The ChR2V gene was introduced into the EcoRI and HindIII sites of the 6P1 plasmid. The synapsin promoter was exchanged for a hybrid cytomegalovirus enhancer/chicken beta-actin promoter, and AAV-ChR2V was constructed. The pAAV-RC and p-Helper plasmids were obtained from Stratagene (La Jolla, CA, USA). High titer (1–10×10^12^ particles/mL) rAAV vectors (rAAV-ChR2V) were purified using the method of Auricchio et al [24].

### Stereotactic surgery (placement of cannula, optic fiber and electrodes, and injection of viral vector)

Animals were anesthetized with ketamine and xylazine (80 mg and 8 mg/kg, i.p.). Lack of responsiveness was checked by toe pinch every 15 min. Rectal temperature was monitored and maintained at 37.0°C. After a midline scalp incision, burr holes were drilled in the skull over the hippocampal formation and/or sensory-motor cortex. The dura was carefully removed, and optic fiber and/or electrodes were lowered at the coordinates defined in each experiment. A stainless-steel screw was driven into the bone above the contralateral cerebellum and served as ground and recording reference. The coordinates and depth of optic fiber-electrode insertion was determined relative to bregma as (anterior-posterior (AP), −3.6 mm; medial-lateral (ML), 2.5 mm: dorsal-ventral (DV), −2.5 mm), (AP, −5.4 mm; ML, 5.0 mm; DV, −7.0 mm), (AP, −1.8 mm; ML, 1.5 mm; DV, −4.0 mm), (AP, −3.0 mm; ML, 4.5 mm; DV, −7.5 mm) and (AP, 2.0 mm; ML, 3.0 mm; DV, −1.0 mm), respectively for the septal hippocampal formation, temporal hippocampal formation, anterior thalamus, amygdala, and sensorimotor cortex. The coordinates for viral vector injection was AP −3.6 mm, ML 2.5 mm, DV −3.5 mm. The location of fiber/electrode was confirmed histologically. AAV vector infusion was performed using a micro-infusion method as described previously [25].

### Photostimulation and electrophysiological recordings

Simultaneous photostimulation and electrical recording were accomplished by using a hybrid optical fiber with electrode (custom-made). A platinum lead and optic fiber (80-µm diameter) was accommodated in an electrically-isolated cannula with a 260-µm diameter. The fiber-optic cable was attached to a 451 nm-diode pumped laser (Optohub, Inc., Saitama, Japan), which emitted 1.2–25.0 mW of light at the tip of the fiber. Stimulation was controlled by Powerlab (AD instrument, Inc., Lexington, Australia). Electrophysiological signals were amplified and band-pass-filtered (1000-fold, band-pass 0.07 and 10 kHz) and digitized at 10 kHz by a Multichannel Acquisition Processor (Plexon, Inc., Dallas, TX, USA). Electromyography of the brachial muscles was also simultaneously recorded in a part of experiment. Location of optic fiber/electrode was verified histologically in hematoxylin-eosin stained sections.

### c-Fos expression after induced seizure-like afterdischarges

Next, we investigated the brain regions activated by the induced seizure-like afterdischarges. The septal hippocampus of *ChR2V*-TG rats was photostimulated once, and the brain was removed 1 hour later. Two different parameters were used, one for afterdischarge induction (*n* = 9 rats) and the other for no afterdischarge induction (control, *n* = 6 rats). Stimulus parameters were 20 mW, 10 Hz, 10 ms pulse width (duty ratio 0.1), and 10 s duration for the former and 20 mW, 0.5 Hz, 10 ms pulse width, 200 s duration for the latter. The 0.5 Hz stimulation was very much below the threshold for afterdischarge induction according to our results. Note that the total durations of photo-pulses were same between two conditions. In the control case, additional pulse photostimulation (20 mW, 10 Hz, 10 ms pulse width, 10 s duration) was performed to induce afterdischarges immediately before brain removal to confirm that the stimulation was appropriately applied to the hippocampus. This control stimulation was not expected to affect c-Fos signals due to its delayed expression profile. c-Fos expression, a surrogate marker of neuronal excitation, was analyzed by immunohistochemical staining.

Rats were deeply anesthetized with a lethal dose of pentobarbital (80 mg per kilogram of body weight, i.p.) and transcardially perfused, first with phosphate buffered saline (PBS) and then with 4% paraformaldehyde. Following perfusion, brains were immersed in 4% paraformaldehyde for 24 h, soaked for 2–3 days in 100% alcohol, cleared in xylene, and embedded in paraffin. Serial coronal brain sections (5 µm) were sectioned on a microtome and labeled with a rabbit polyclonal anti-c-Fos antibody (1∶1,000; Santa Cruz, Inc., Santa Cruz, CA, USA), which was detected by the fluorescent secondary Alexa 568 goat anti-rabbit antibody (1∶200; Invitrogen, Carlsbad, CA, USA). Sections were then mounted and coverslipped using Vectashield with DAPI (Vector Laboratories, Inc., Burlingame, CA, USA). Gene-expression efficacy was quantified by counting the number of c-Fos positive cells divided by the number of DAPI-positive cells under a microscope (BZ-9000, Keyence, Inc., Osaka, Japan).

The proportion of c-Fos positive cells to total cells (% c-Fos positive cell) was counted in the granule cell layer of the dentate gyrus (DG) and in the pyramidal cell layer of CA3 and CA1 on the septal and temporal sides of the stimulated hippocampal formation. Cell counting was performed on a coronal section made for the septal and temporal hippocampus. The section was chosen by reference to the Watson-Paxinos's rat brain atlas; AP – 3.6 mm for the septal hippocampus and AP – 5.4 mm for the temporal hippocampus [26]. The former was made adjacent to the optic fiber insertion. The latter included the appearance of the ventral hippocampus. One section was analyzed per animal, each for the septal and temporal hippocampus. The ventral aspect was used for counting in the temporal hippocampus. The counting was performed offline on the images fused from the epifluorescence. Images were reviewed and scored at 200-fold magnification on two different fields of view, each for DG, CA3 and CA1 region. At least 200 granule cells and/or pyramidal cells were counted by two independent observers (SO, MI) and averaged.

### Spatio-temporal dynamics of seizure-genesis along longitudinal hippocampal axis

To investigate the spatio-temporal dynamics of seizure-genesis, we performed multisite LFP recording during seizure-like afterdischarge induction. For this experiment, 10 Hz pulse stimulation was delivered for 30 seconds at a 10 ms pulse width (duty ratio 0.1) and a 2.7–2.8 mW light intensity at the tip of fiber. A linear-array multicontact electrode (16 contacts with 150-µm spacing, U-probe, Plexon Inc., Dallas, TX) was inserted into the dentate gyrus-to-hilus of the temporal hippocampal formation parallel to the longitudinal axis. LFPs were recorded during and after pulse photostimulation of the septal hippocampus (10 afterdischarges per rat, *n* = 3 rats, total 30 afterdischarges). Lower light intensity was used to reduce the amplitude of photostimulus-evoked potentials. This was aimed to enhance detection of correlations in spontaneous activities between regions. Longer stimulation duration was attempted to induce afterdischarges reliably in low light intensity. To see the causal relationship and synchrony between locations along the septo-temporal (ST) orientation of the hippocampus, the Granger causality (GC) and coherence of the LFPs was calculated between electrode pairs. Although LFP signals should have nonlinear relationships, it is very difficult to infer concrete nonlinear relationships from time-series. We focused only on the linear part of the relationships in this study, so that linear measures, the Granger causality and coherence, were employed.

Additional experiment was performed under two simultaneous recording from the septal and the temporal side of the hippocampus using single electrodes. In one situation, the photostimulation was applied in the septal side of the hippocampus to induce seizure-like afterdischarges (*n* = 3 rats, total 17 afterdischarges). In the other situation, the stimulation was applied in the temporal side of the hippocampus to induce seizure-like afterdischarges (*n* = 3 rats, total 18 afterdischarges). 10 Hz pulse stimulation was delivered for 30 seconds at a 10 ms pulse width (duty ratio 0.1) and a 2.7–2.8 mW light intensity at the tip of fiber. The GC was calculated between two electrodes.

### LFP analysis

#### Preprocessing

The elecrophysiological signals were band-pass-filtered between 1 and 300 Hz and down-sampled to 1 kHz for later analysis. All the following calculations were performed in MATLAB version 15 (MathWorks, Inc., Natick, MA).

#### Time-frequency analysis

The time-frequency representation of the LFP power was obtained by the wavelet transformation, with the Gabor mother wavelet defined by
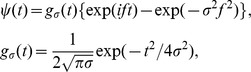
where 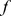 is the central frequency, and 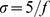.

Peak frequency was defined in the time-frequency distribution as the frequency with maximum power.

#### Granger causality, Granger index, and coherence

Degree of information flow was evaluated by Granger causality (GC) [27], [28], where 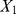 and 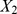 are two zero-mean stationary signals whose time observations are 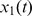 and 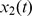, with 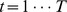. If the temporal dynamics of 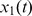 and 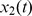 could be written by an univariate autoregressive model of order 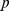, the model would be
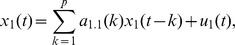


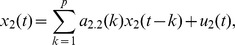
where prediction error 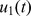 and 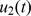 for a signal depend only on its own past. 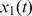 and 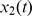 can be assumed to be represented by the following multivariate auto-regressive model of order 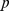,
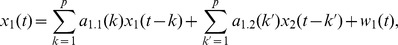


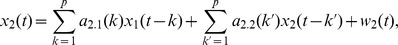
where prediction error 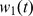 and 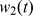 depend on the past of the two signals. So in the model above, if 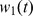 were smaller than 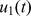, then we could conclude that 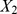 caused 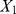. Similarly, if 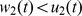, then 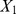 caused 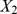. In short, if knowing time series 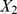 helped to predict the future of the other time series 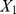 and 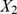 “Granger caused” 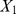. The magnitude of the GC was quantified by the log-ratio of the prediction error variance,
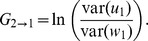
The model order was chosen using Akaike's information criterion (AIC) and Baysian information criterion (BIC).

Dominance of the information flow, from septal side to temporal side or its opposite direction, was evaluated by an index, we called the “Granger index,” defined by
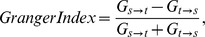
where 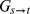
and 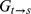 denote the GC from septal to temporal sides and the GC from temporal to septal sides, respectively. The Granger index, 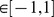, was positive if 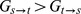, otherwise it was negative. GC indicates positive values when the septal-to-temporal causality is larger than the opposite direction. Otherwise, it is negative.

We recorded the LFPs using a 16-channel multi-site probe, so we had 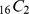 LFP-pairs. To assess micro-scale causal relationships, widely separated LFP-pairs were excluded for Granger causality and Granger index. Only pairs whose distance was 450 µm or less (i.e., three times the inter-electrode interval or less) were used in calculation of the mean and the variance of the Granger causality, and the Granger index.

Coherence was introduced to evaluate the synchronization of signals in the frequency domain. The coherence of signals 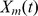 and 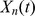 was calculated as follows,
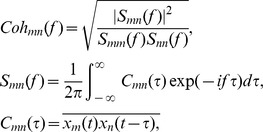
where overline 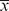 represents a temporal averaging operation, and 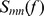 and 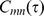 are the cross-spectrum and cross-correlation, respectively. The coherence is essentially the square of the correlation coefficient between the corresponding frequency component of 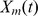 and 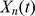.

In this study, we define the coherence by the average across frequencies:
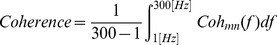
For coherence, all the LFP-pairs were used for calculation of the mean and variance. The coherence quantifies the degree of synchrony in LFPs; 0 indicates complete asynchrony and 1 indicates complete synchrony.

#### The moving window

The GC and coherence were calculated using a 1000-ms-length moving window. The moving window moved in 100-ms time steps, so each window overlapped by 900 ms. The large overlapping was employed to draw the behavior of the GC and coherence in a sub-second temporal resolution. The center of the moving window moved from −10 seconds to 100 seconds around stimulus onset.

#### Transition on a state-space

The scatter plot clusters (Granger-index, coherence) were calculated by the k-means clustering method (k = 3 in this study) [29].

## Results

### Seizure-like afterdischarges were induced by repetitive photostimulation of the ChR2-expressing hippocampus

**Figure 2:**
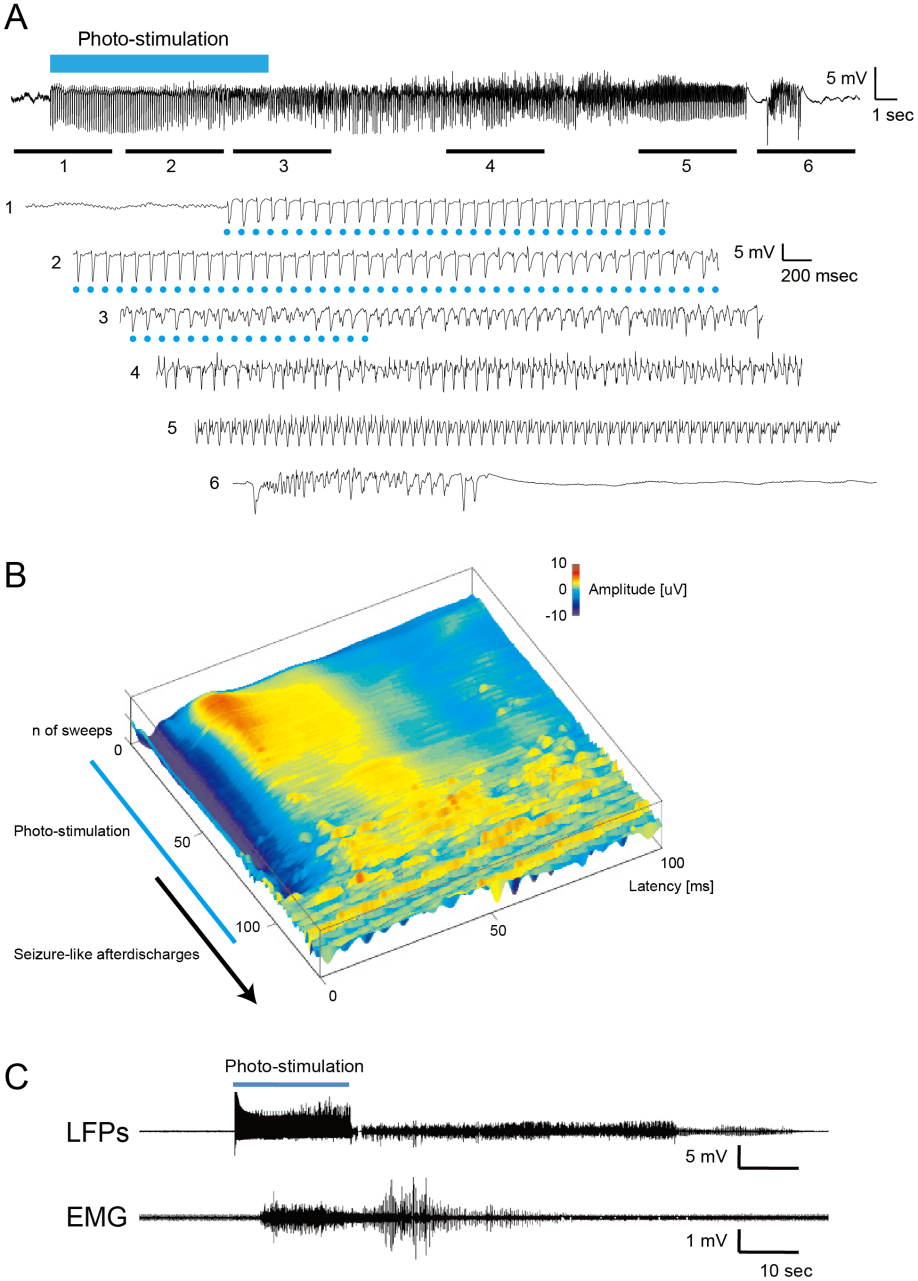
Electrical activities of optogenetically induced seizure-like afterdischarges. (A) Example traces of local field potentials recorded at the site of stimulation. Repetitive photostimulation at a 10 Hz frequency and a 0.05 duty ratio was applied to the hippocampus for 10 seconds by a hybrid-electrode fused with an optic fiber. The entire recording of the photostimulation and induced seizure-like afterdischarge is presented at the top. Magnified views below corresponded to the numbers. Blue dots indicate photostimuli. At the start of stimulation, only evoked potentials followed each photo pulse (1). Spontaneous activities, which were not time-locked to stimuli, emerged in addition to the evoked potentials (2) and persisted after the stimulation ended (3). The activity gradually became high in amplitude and rhythmic (4,5) then stopped spontaneously (6). Note that waveform changes were observed even under stimulation (especially in A2). (B) Raster presentation of LFPs during and immediately after stimulation demonstrates that evoked responses are gradually replaced by non-time-locked activity. (C) Simultaneous recording of optogenetically induced afterdischarges and electromyogram (EMG) of the contralateral forelimb. Clonic EMG activities appeared during stimulation, but never persisted after the LFP afterdischarge.

Seizure-like afterdischarges were successfully induced and reproduced by repetitive photostimulation to the hippocampus (Figure 2A). No rats died during the experiment. Seizure-like afterdischarges observed in this study were characterized as follows. During the initial stimulation phase, only evoked potentials followed each photostimulus pulse. High-amplitude spontaneous activity, which was not time-locked to stimuli, emerged in addition to the evoked potentials during stimulation and gradually became rhythmic and dominant (Figure 2B). This rhythmic activity self-persisted after the stimulation ended and spontaneously ceased in 8.4–85.5 (39.8±20.4; *n* = 115) seconds. The afterdischarge duration was independent from stimulus frequency and duty ratio (two-way analysis of variance [ANOVA], p = 0.5). The seizure-like afterdischarge was associated with clonic twitches in the whiskers and paws and tonic conversion of the tail. Simultaneous LFP-electromyogram recordings revealed activation of brachial muscles, which never started earlier than afterdischarge (Figure 2C).

**Figure 3:**
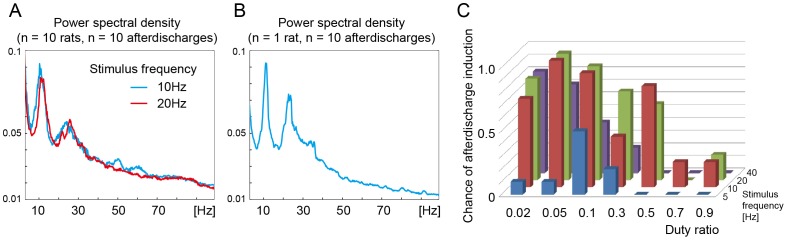
Characteristics of optogenetically induced seizure-like afterdischarges. (A) Average curve of frequency spectrum for the first 10 seconds of the induced afterdischarge after photostimulation. Seizure-like afterdischarges possessed frequency peaks around 10 and 25 Hz similarly after 10 Hz (blue) and 20 Hz (red) photostimulation. The duty ratio was 0.05 in boths. The power spectral density curve was an average of 10 afterdischarges recorded from 10 rats. This also indicates that the frequency characteristic was common across animals. (B) Average curve of frequency spectrum of the first 10 seconds of afterdischarges recorded in a single animal. The power spectral density curve was an average of 10 afterdischarges including 4, 5, and 1 afterdischarges after 10, 20 and 40 Hz photostimulations respectively with various duty ratios. Seizure-like afterdischarges showed frequency peaks around 10 and 25 Hz. (C) Chance of afterdischarge induction is plotted against stimulus frequency and duty ratio (*n* = 115 seizures, *n* = 10 rats). The highest chance was 1.0 (10 afterdischarges induced in 10 trials) observed with 10 and 20 Hz stimulus frequencies and a duty ratio of 0.05. Optical intensity was 19–22 mW at the tip of optic fiber and stimulus duration was 10 seconds.

The self-sustained afterdischarges possessed frequency peaks around 10 and 25 Hz similarly after 10 Hz and 20 Hz photostimulation (Figure 3A). This frequency characteristic was common across as well as within animals (Figure 3B).

The chance of afterdischarge induction was dependent on the stimulus parameters. The highest rate was 1.0 (10 of 10 trials) observed at a 10 and 20 Hz pulse frequency and a smaller duty ratio of 0.05 (Figure 3C). Continuous stimulation did not efficiently induce afterdischarge (induction ratio = 0.1; data not shown). No clear refractory or facilitatory effect was observed on occurrence of afterdischarges in the 5 minutes interval stimulations. The number of induced afterdischarges was not statistically different between the first 10 trials and the last 10 trials (paired t-test, p = 0.425).

**Figure 4:**
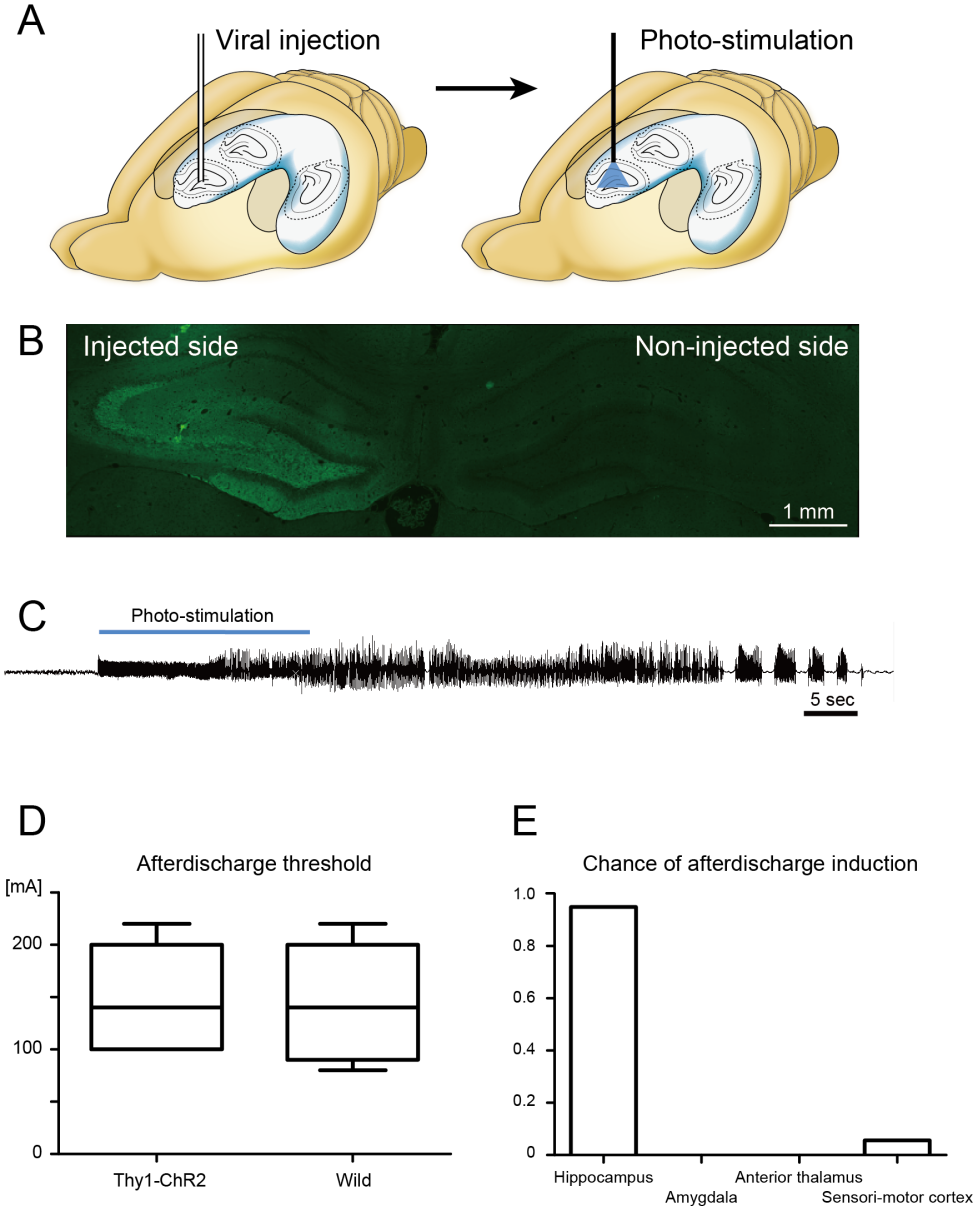
Afterdischarge induction was not due to an innate susceptibility of *ChR2V*-TG animals to seizures. (A) AAV5-ChR2V viral vector was injected to rat hippocampus 4 weeks prior to the afterdischarge induction experiment (*n* = 3). (B) Fluorescent images of ChR2V protein (green) expression in fibers running through the hilus, the molecular layer of the dentate gyrus, and the striatum radiatum of CA3. (C) Raw waveform recorded in the hippocampus during afterdischarge induction. Repetitive photostimulation induced seizure-like afterdischarge similar to that observed in the experiment using *ChR2V*-TG rats. At least one afterdischarge was induced after photostimulation on any combinations of stimulus frequency (5, 10 ,20 Hz) and duty ratio (0.05, 0.1, 0.2). Optical intensity and stimulus duration were fixed at 19–22 mW at the tip of optic fiber and at 20 seconds, respectively. (D) Electrical stimulation was delivered to induce “classical” afterdischarge in the hippocampus. No significant difference was observed in the afterdischarge threshold between *ChR2V*-TG rats (*n* = 5) and wild-type Wistar rats (*n* = 5), suggesting that no inherent excitability existed in the transgenic rats. (B) Repetitive pulse photostimulation was delivered to the hippocampus and extra-hippocampal structures in *ChR2V*-TG rats (*n* = 4). The chance of afterdischarge induction was very low or zero in the amygdala, anterior thalamic nucleus and sensorimotor cortex. Stimulation parameters were set at 10 Hz with a duty ratio of 0.05. Optical intensity and stimulus duration were fixed at 19–22 mW at the tip of optic fiber and at 10 seconds, respectively. It should be noted that seizure-like afterdischarge was induced one out of 10 trials after the stimulation of the sensori-motor cortex.

Afterdischarge induction was not due to an innate susceptibility of *ChR2V*-TG animals to seizures. Repetitive photostimulation to the hippocampus of wild-type Wistar rats transfected with the rAAV-ChR2-Venus vector successfully reproduced seizure-like afterdischarges (Figure 4A–C). At least one afterdischarge was induced after photostimulation on any combinations of stimulus frequency (5, 10, 20 Hz) and duty ratio (0.05, 0.1, 0.2). The afterdischarge threshold was not significantly different (144±57.3 and 148±52.2 µA) between *ChR2V-TG* rats and wild type Wistar rats (*n* = 5, each) (Figure 4D).

Optic fiber insertion and photostimulation did not induce afterdischarges, by itself, in the absence of ChR2. No afterdischarges were observed after repetitive pulse photostimulation of the “normal” hippocampus in wild-type Wister rats. The chance of afterdischarge induction was significantly lower following stimulation of extra-hippocampal structures than of hippocampus in *ChR2V-TG* rats (*p*<0.01, chi square test) (Figure 4E). It should be noted that seizure-like afterdischarge was induced one out of 10 trials after the stimulation of the sensori-motor cortex.

### Induced seizure-like afterdischarge activated the entire hippocampus

**Figure 5:**
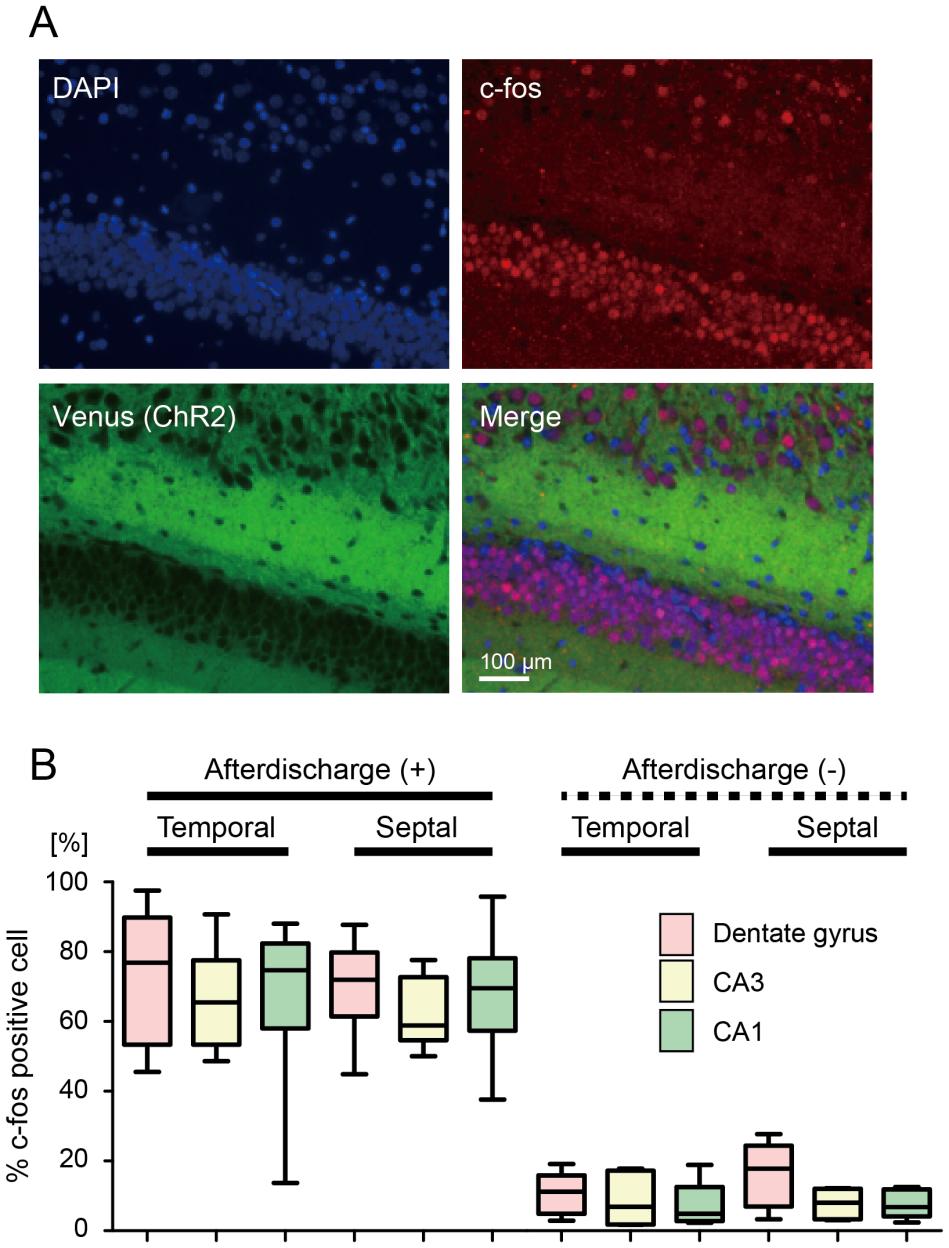
Optogenetic seizure-like afterdischarges activated the entire hippocampus. (A) Fluorescent images of dentate-hilar neurons expressing ChR2V and labeled for the immediate early gene product c-Fos show robust neuronal activation produced by seizure-like afterdischarge. (B) The proportion of c-Fos positive neurons to total cells was significantly higher in the afterdischarge-induced hippocampus (*n* = 9) than in the control group (*n* = 6). No region-specific increases were observed.

The c-Fos expression was induced in the entire hippocampus of *ChR2V*-TG rats after a single seizure-like afterdischarge induced by pulse photostimulation (Figure 5A, Figure S1). % c-Fos positive cell was significantly higher in the afterdischarge group than in the no-afterdischarge group (two-way ANOVA, *p*<0.0001). No significant differences were observed in the % c-Fos positive cell between septal and temporal hippocampus (*p* = 0.90) (Figure 5B). The c-Fos expression was proportional among hippocampal subregions in seizure group (*p*<0.01 in CA3 vs. DG, *p* = 0.043 in CA3 vs. CA1) (Figure S2A, B).

### Bidirectional and asymmetric causal relationships were observed along the septo-temporal axis of the hippocampus during seizure-like afterdischarges

**Figure 6:**
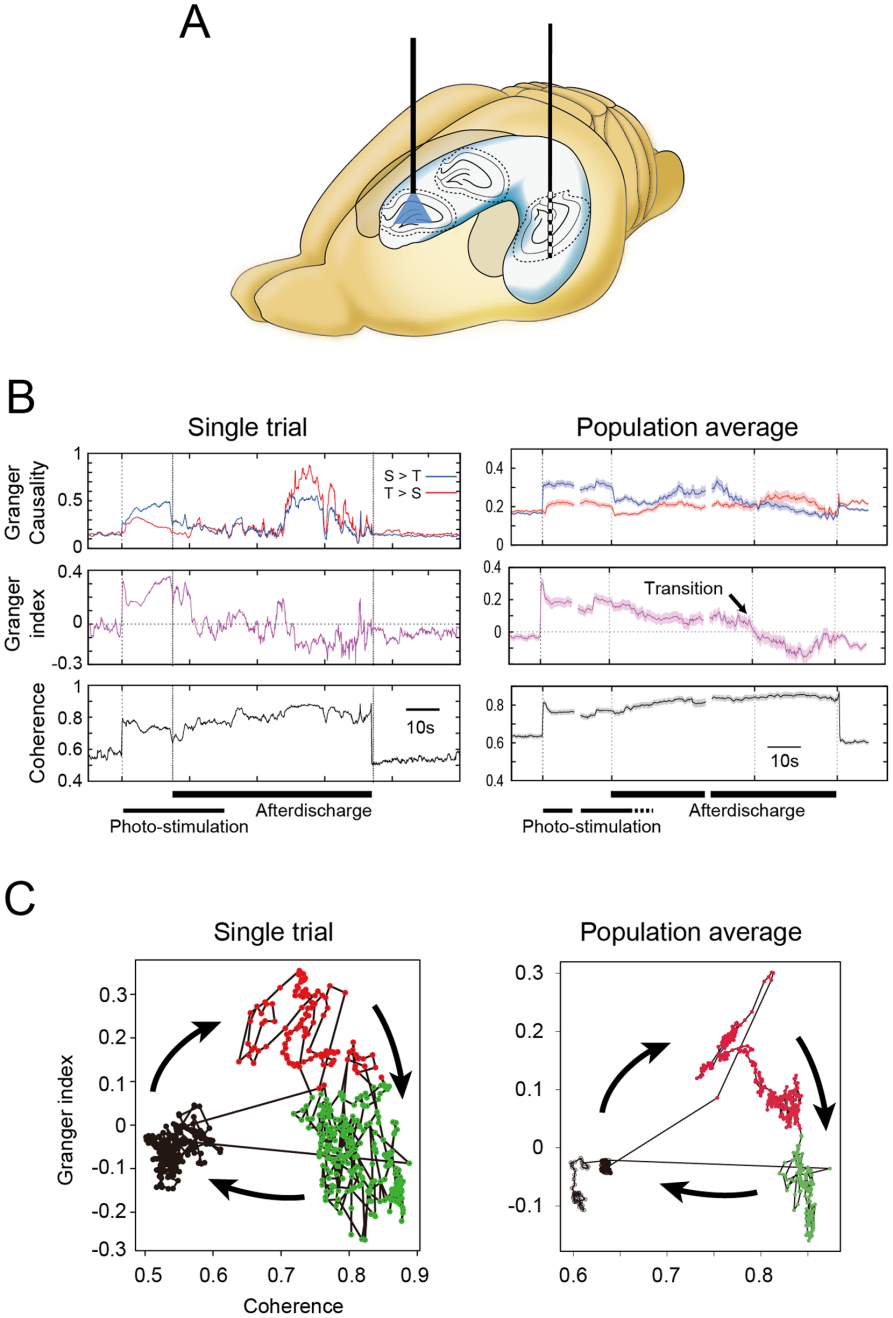
Causal relationships and coherences were dynamically changed along the septo-temporal axis of the hippocampus during seizure-like afterdischarges. (A) Pulse photostimulation was delivered to the septal hippocampus, and induced afterdischarges were simultaneously recorded from multi-contact electrodes inserted along the septo-temporal axis of the temporal hippocampus (*n* = 3 rats). (B) Time courses of Granger causality, Granger index, and coherence are shown for both a single induced afterdischarge and a population average of afterdischarges (10 afterdischarges per rat, total 30 afterdischarges). On the population average, the thick line and shaded areas indicate the mean and 99.9% confidence interval, respectively. The average and standard deviation were calculated from the recorded LFPs of 30 trials of three rats (total 1620 LFP-pairs). The Granger causality increased in both directions but was greater in the septo-temporal direction at afterdischarge initiation. Temporo-septal causality became higher toward the end of the afterdischarge, causing transition of the Granger index to a negative value. Coherence was gradually increased toward the end of the afterdischarge. (C) State-space plots of Granger index and coherence. The population average (right) shows the mean of 30 afterdischarges. K-mean clustering (k = 3) revealed three distinct states: 1) resting state in which causality and synchrony were both low (black); 2) early phase of afterdischarge characterized by dominant septo-temporal causality and increase in coherence (red); and 3) late phase of afterdischarge characterized by reversal of causality index to the temporo-septal direction and increase in coherence (green). Transitions between phases are indicated by arrows.

Multisite LFP recording revealed that induced seizure-like afterdischarges were characterized by an increase in the GC along the ST hippocampus (Figure 6A). During hippocampal stimulation, the GC indices increased in both the septal-to-temporal and temporal-to-septal directions, but were higher in the former (Figure 6B, top panels). The Granger index quantified the dominant direction (Figure 6B, middle panels; see Methods for details). The Granger index is positive if the septal-to-temporal is the dominant direction. Otherwise it is negative. In the course of self-sustained afterdischarges after stimulation ended, the septal-to-temporal GC gradually decreased to the same level as the temporal-to-septal GC. Toward the final phase, the GC indices again increased in both directions, and the temporal-to-septal GC became higher in comparison. The seizure-like afterdischarge was also characterized by an increase in coherence (Figure 6B, bottom panels). Zero indicates complete asynchrony and 1 indicates complete synchrony of LFPs. The coherence showed a gradual increase toward the end of the afterdischarge and vanished at afterdischarge termination. An example of GC and coherence matrix was presented in Figure S3. Granger causality did not depend on the distance between electrode pair, but rather on their relative position. Increase of the septal-to-temporal causality tended to occur in electrode pairs in the septal side, while increase of the temporal-to-septal causality did in the temporal side. Higher coherence was seen in closer pairs of electrodes.

**Figure 7:**
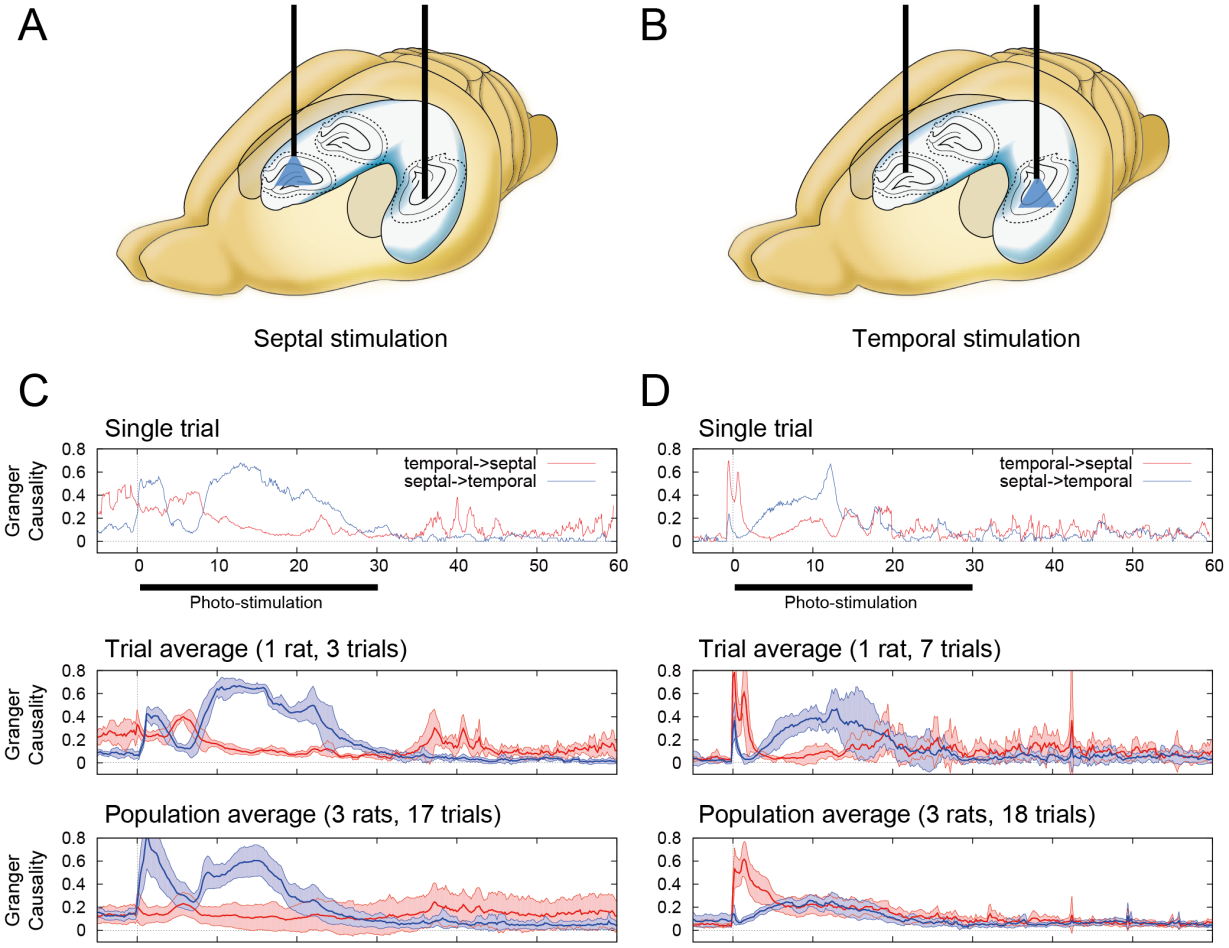
Causality analysis of LFPs in S-T axis of hippocampus in septal and temporal stimulations. (A,B) Schematic of experimental set-up used for two simultaneous recording and the septal and temporal hippocampal photostimulation (*n* = 3, total 17 seizures). (C, D) Traces of bidirectional Granger causality (GC) scores from recorded LFPs in the stimulation to the septal (C) and temporal (D) hippocampus. (Top panels) Example traces of the GC scores. (Middle panels) Average traces of bidirectional GC scores, among three trials in one rat (septal stimulation) and seven trials of one rat (temporal stimulation). Colored regions indicate 95% confidence interval. (Bottom panels) The same as the middle panels, but the average traces were calculated using 17 trials of three rats (septal stimulation) and 18 trials of three rats (temporal stimulation). Increase of septal-to-temporal GC was observed both during the septal and temporal stimulations.

The above changes in GC were not a simple propagation of activity from the stimulation site to the other. Increase of septal-to-temporal GC was observed both during the septal and temporal stimulations in the additional experiment with two simultaneous recording (Figure 7).

A state-space plot between the Granger index and coherence indicated the presence of at least three attractors by the k-means clustering (Figure 6C): 1) a resting state before and after the afterdischarge; 2) afterdischarge initiation with moderate coherence and dominant septo-temporal GC; and 3) afterdischarge termination with increased coherence and dominant temporo-septal GC. These changes were present across animals.

## Discussion

Repetitive pulse photostimulation to the ChR2-expressing rodent hippocampus reproducibly induced seizure-like afterdischarges, which was self-sustained and propagated through the entire hippocampus. This phenomenon was confirmed in both *ChR2V*-TG rats and wild-type Wistar rats transfected with AAV5-ChR2V vectors. Afterdischarge induction was most efficient at 10 and 20 Hz stimulus frequencies with a smaller duty ratio, and the induced afterdischarges were stereotypical in terms of frequency characteristics and duration. Simultaneous recording of multisite LFPs revealed bidirectional and asymmetric causal relationships along the ST axis of the hippocampus during genesis and termination of seizure-like afterdischarges. State-space representation of causality and coherence indicated three discrete states of the phenomenon: 1) resting state; 2) afterdischarge initiation with moderate coherence and dominant septo-temporal causality; and 3) afterdischarge termination with increased coherence and dominant temporo-septal causality.

### Optogenetics for a novel model of seizure-like afterdischarges

The current study proposes a novel optogenetic methodology to induce seizure-like afterdischarges in *vivo* animals with photosimulation to the local hippocampus circuits. Epilepsy is one of the most common neurological disorders that can involve any part of the central nervous system [30], and the hippocampus is a major site that generates focal seizures in humans, particularly in those with drug-resistant epilepsy [4]. Although most previous studies employed optogenetics for precise and specific control of neurons, here, we applied the technique to perturb the hippocampal network and subsequently reproduce a disease state.

Our novel method of afterdischarge induction is advantageous in its reproducibility, low mortality (almost zero) and artifact-free electrophysiological observations, compared with conventional models of seizures. Repetitive electrical stimulation of the brain has long been known to induce “epileptiform” discharges, especially in the hippocampus [11], [31], [32]. Here we applied optogenetic approach instead of electrical stimulation for seizure induction. To the best of our knowledge, optogenetics have not been used for development of a model of epileptic disorders. The advantage of our approach is artifact-free observation of electrophysiology during stimulation. LFPs were successfully recorded and analyzed during stimulation to reveal spatio-temporal dynamics of seizure-like afterdischage. The artifact-free electrophysiological recording during stimulation provides a valuable opportunity to study neurophysiological processes of seizure genesis.

Many other acute seizure models have been developed by altering cortical excitability, such as topical application of penicillin, systemic or local administration of kainic acid [6]. The advantage of electrical or optogenetic stimulation is replication of seizures without minimum histological damages. Ten or more seizure-like afterdischarges can be reproduced in one rat without mortality using this model. The model is suitable to investigate neural networks that maintain seizure activity and secondary effect of epileptic seizures.

It should be noted here that this is a model of epileptic seizures, rather than epilepsy. Models of epilepsy, the model that spontaneously generates epileptic seizures, would be more relevant to study epileptogenesis [6], [9]. However, a model of epileptic seizures is still important in understanding its mechanisms of genesis, because many current therapeutics are developed to suppress or counteract epileptic seizures [7]. Efficient induced-seizure model is applicable for evaluating the efficacy of epilepsy therapeutics [33]. Anti-seizure efficacy could be measured as a probability of afterdischarge induction in a short time.

Currently it is difficult to determine what subpopulation of hippocampal neurons was activated to trigger seizure-like afterdischarges in this experiment. In our model, ChR2 was expressed non-selectively both in pyramidal neurons and interneurons. According to previous reports, penetration depth of light would range in an order of hundred µm or 1 mm [34], [35]. Theoretically, the optical stimulation activation threshold depends on ChR2 channel density and surface area [36], implying that optical stimulation mainly acts on cell body, but also on passing fibers to some extent. Besides pyramidal neurons, interneurons with higher input resistance may respond more easily to ChR2 depolarization. One previous study showed that optogenetic stimulation of the mouse neocortex expressing ChR2 could produce the evoked LFP similar to electrical stimulation [37]. Thus, it is possible that subsequent activation process would be similar between optogenetic and electrical stimulation. To further understand mechanisms of seizure-genesis, advanced strategy is necessary, including selective introduction of ChR2 to excitatory neurons or to inhibitory neurons, use of hyperpolarizing channels such as Halorhodopsin (NpHR), and anatomically selective application of photostimulus.

### The septo-temporal network is important in hippocampal seizure-genesis

Seizure genesis is characterized by its diffuse and dynamic nature. When observed using LFPs, seizure activity is initiated diffusely or focally in different locations in the hippomcapus, and the onset patterns can be different among seizure in a single subject [10]. Once initiated, focal seizure activity is propagated along not only the transverse but also the septo-temporal (longitudinal) direction of the hippocampus, and the propagation pattern can be bidirectional in a single seizure [38].

Importance of the septo-temporal connections in seizure propagation has already been investigated [38]–[40]. Derchansky et al examined latencies of waveform peaks in multi-site LFPs along the septo-temporal axis of hippocampus during seizures using low-magnesium model and focal tetanic stimulation model of isolated hippocampus [38]. In the low-magnesium model, epileptiform discharges always started from ventral hippocampus, but propagation direction can “flip” several times in later course of seizures. In the focal tetanic stimulation model, epileptiform discharges always propagated from the stimulation site to the other, and similar bidirectional propagation followed. Interestingly, they noted the flip pattern can be independent between low (<10 Hz) and high frequency components. Our results further support those previous data on the septo-temporal interaction in seizure genesis. We showed bidirectional changes in information flow during seizure-like afterdischarges similarly with Derchansky's study, while our study used in *vivo* model and different measures. Using Granger causality, we showed that the septo-temporal interaction was not a simple propagation of the activity from the stimulation to the other. Both in the septal and temporal stimulations, increase of septal-to-temporal GC was observed during afterdischarges.

Long-range direct connection is relatively scarce along the hippocampal septo-temporal axis, in spite of the potential role in seizure dynamics. Filipe et al studied septo-temporal propagation of afterdischarges caused by electrical stimulations [39]. They investigated both linear and non-linear measures for association, and the value of association was generally larger in non-linear than liner method. Larger association was found between ipsi- and contra-lateral septal hippocampus than between the septal and temporal hippocampus. Relatively low association noted in Filipe's study is probably explained by long distance between recording sites for comparison, i.e. septal and temporal hippocampus. In such situation, they found that non-linear measures were better than linear ones to detecting associations. Liner association was better investigated in short-range comparison using multi-site LFP recording, like in our study and Derchansky's report. This implicates that long and direct association fibers are relatively few in the septo-temporal direction compared with commissural connections [39].

Behaviors of dynamic and highly interacting systems, such as the central nervous system (CNS), are investigated from the viewpoint of non-linear complex systems [41]. Occurrence of epileptic seizures has been interpreted as a transition between bi- or multi-stable states of neuronal networks (i.e., normal or “inter-ictal” states versus epileptic or “ictal” states) [42]. The theory of non-linear dynamics has frequently been applied to the human electroencephalogram (EEG) and used as an early warning sign before seizure transition [42], [43]. The theory is also used for better understanding the spatio-temporal dynamics of seizure [43], [44].

**Figure 8:**
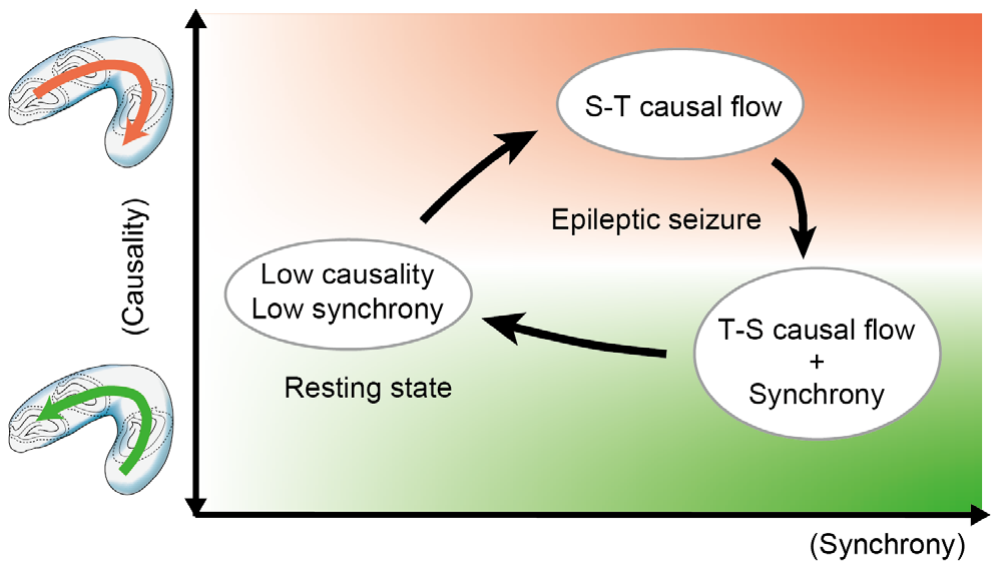
State transitions during hippocampal seizure-like afterdischarges. Three discrete states are illustrated by means of causality and coherence. Bidirectional networks along the longitudinal hippocampus work hierarchically in the genesis and termination of seizure-like afterdischarges.

Bidirectional causal relationships revealed by GC analysis suggest the presence of hierarchical network dynamics along the longitudinal axis of the hippocampus in generating, maintaining and terminating seizures. The GC analysis was previously applied to an absence epilepsy model, in which the thalamo-cortical recurrent network is responsible for seizure genesis. A bidirectional increase in causality was observed during seizures, but the intensities and time courses of the two causalities were asymmetrical (i.e., the thalamo-frontal causality was more intensive, and the fronto-thalamo causality was restored to the initial level before seizure cessation) [45]. Two classes of network associations were hypothesized: “driving” and “modulating” connections during seizures based on the “no-strong-loop” hypothesis of recurrent neuronal networks [46]. Bidirectional causal relationships in epileptic seizures were also demonstrated in the lamellar organization of the hippocampal network. The GC from the CA1 subfield to the dentate gyrus was increased prior to and during seizure, and causality reversal occurred before seizure cessation [44]. The bidirectional causal relationships observed in our study suggest the presence of hierarchical and asymmetrical connections along the longitudinal hippocampal network, which may be engaged in seizure genesis (Figure 8) [43], [44].

From an anatomical standpoint, the hippocampal cell population and their synaptic connections are not symmetrical between subregions along the longitudinal axis [47]. Packing densities of granule cells and CA3 pyramidal cells vary along the ST axis [48], suggesting that the hippocampal longitudinal circuit is not a simple recurrent circuit. Moreover, the septal, intermediate, and temporal parts of the hippocampus display distinctive patterns of connectivity to extra-hippocampal structures [49]. Thus, the above features also support the asymmetrical dynamics of seizures along the ST axis of the hippocampus.

## Conclusions and Perspectives

We developed a novel *in vivo* model of seizure-like afterdischarges in the hippocampus using optogenetics. This novel method of afterdischarge induction enabled simultaneous artifact-free electrophysiological recording, providing a valuable opportunity to study neurophysiological processes of seizures. Moreover, this model is applicable to evaluating the efficacy of antiepileptic drugs or other therapeutics for epilepsy. Anti-seizure efficacy could be measured as a probability of afterdischarge induction in a short time, due to acute and reliable afterdischarge induction with low mortality.

Our findings provide additional evidence for the potential role of the longitudinal hippocampal network in seizure dynamics. Bidirectional networks along the ST axis of the hippocampus are engaged in a hierarchical fashion during generation and termination of seizure-like afterdischarges. A better understanding of seizure dynamics will aid in developing new treatments for epileptic seizures (e.g., selective blockade of the network engaged in seizure genesis).

## Supporting Information

Figure S1 **c-Fos expression in the induced seizure-like afterdischarge.** (A) c-Fos expression in a control rat with no induced afterdischarges. Minimum amount of expression was seen. (B) c-Fos expression at the stimulation site in a rat with induced afterdischarge. (C) c-Fos expression at the temporal hippocampus away from the stimulation site in a rat with induced afterdischarge. Strong c-Fos expression was also seen in the temporal hippocampus.
(TIF)

Figure S2 **Hippocampal subregions are activated in parallel by optogenetically induced seizure-like afterdischarges.** Proportions of neurons expressing the immediate early gene, c-Fos in the afterdischarge-induced group (A) and the control group (B). % c-Fos positive cells in DG and CA1 are plotted against CA3. (A) The level of c-Fos expression in CA3 was correlated with that of DG (*p*<0.01) and CA1 (*p* = 0.043) in the afterdischarge group. (B) The % c-Fos positive cell was low in the control group.
(TIF)

Figure S3 **Examples of the Granger causality and coherence.** Example traces of the Granger causality and coherence (rat 0624, trial 17). The numbers (1 to 16) at the left and upper part of the figure indicate the index of the recording sites on the probe. The site 1 is in the septal side of the hippocampus and nearest to the stimulation site. The site 16 is in the temporal side of the hippocampus and farthest to the stimulation site. The LFPs were recorded from these 16 recording sites (Recording site 2 is broken in this example experiment). The Granger causality and coherence were calculated in all LFP pairs. Right-upper panels are the Granger causality of the LFP pairs. The blue line indicates the causality from septal to temporal direction. The red line indicates the causality from temporal to septal direction. The left-lower panels are the coherence of the LFP pairs. The horizontal axis is time (second) for all panels. The photostimulation was applied from 10 to 40 seconds. Granger causality did not depend on the distance between electrode pairs, but rather on their relative position. Increase of the septal-to-temporal causality tended to occur in electrode pairs in the septal side, while increase of the temporal-to-septal causality did in the temporal side. Higher coherence was seen in closer pairs of electrodes.
(TIF)
