# Supplementary figures and images for: Optogenetically Induced Seizure and the Longitudinal Hippocampal Network Dynamics
Source: PLoS One. 2013 Apr 10;8(4):e60928. doi: 10.1371/journal.pone.0060928 (PMC3622611; doi:10.1371/journal.pone.0060928)

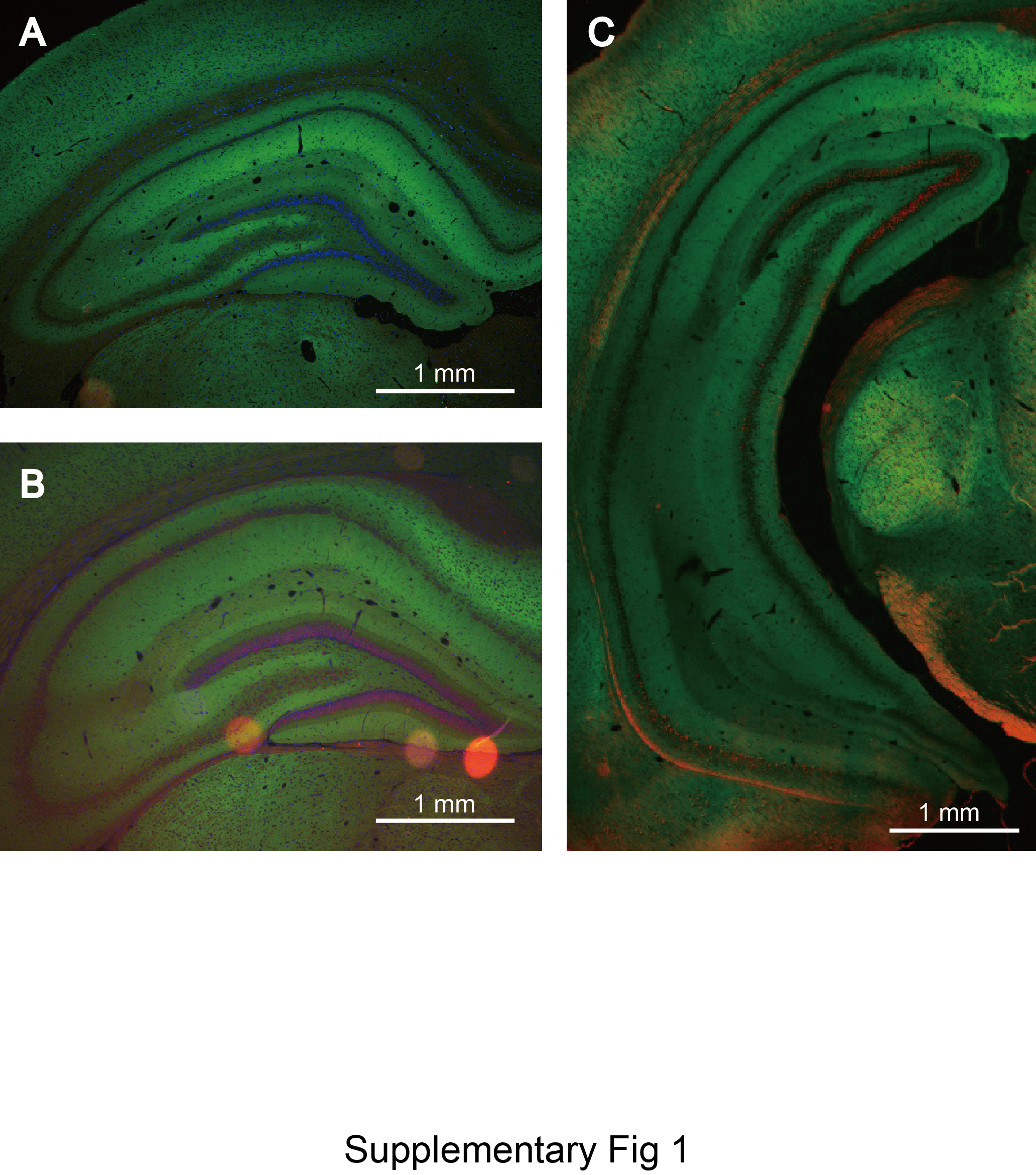

Supplement: Figure S1 — c-Fos expression in the induced seizure-like afterdischarge. (A) c-Fos expression in a control rat with no induced afterdischarges. Minimum amount of expression was seen. (B) c-Fos expression at the stimulation site in a rat with induced afterdischarge. (C) c-Fos expression at the temporal hippocampus away from the stimulation site in a rat with induced afterdischarge. Strong c-Fos expression was also seen in the temporal hippocampus. (TIF) [file pone.0060928.s001.tif]

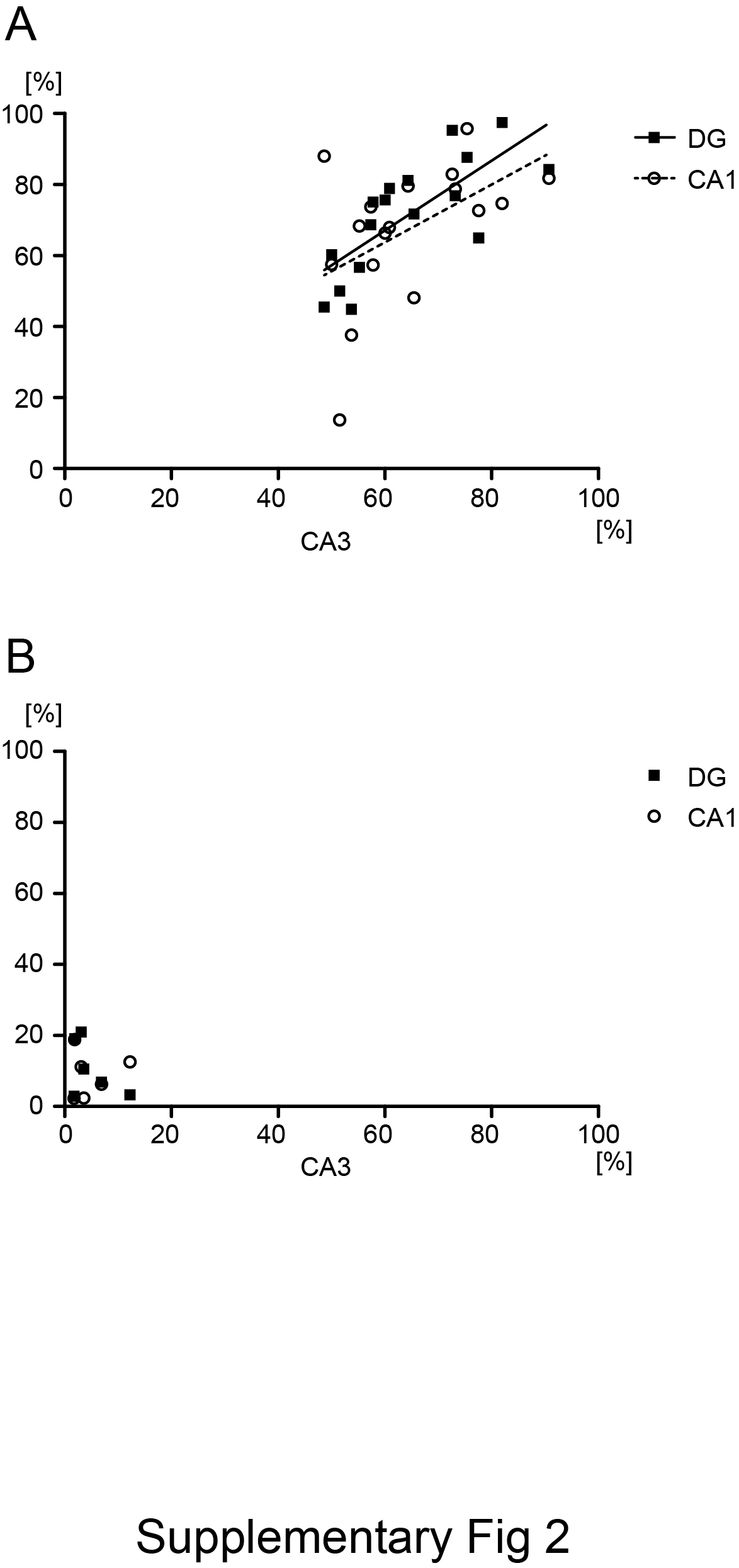

Supplement: Figure S2 — Hippocampal subregions are activated in parallel by optogenetically induced seizure-like afterdischarges. Proportions of neurons expressing the immediate early gene, c-Fos in the afterdischarge-induced group (A) and the control group (B). % c-Fos positive cells in DG and CA1 are plotted against CA3. (A) The level of c-Fos expression in CA3 was correlated with that of DG (p<0.01) and CA1 (p = 0.043) in the afterdischarge group. (B) The % c-Fos positive cell was low in the control group. (TIF) [file pone.0060928.s002.tif]

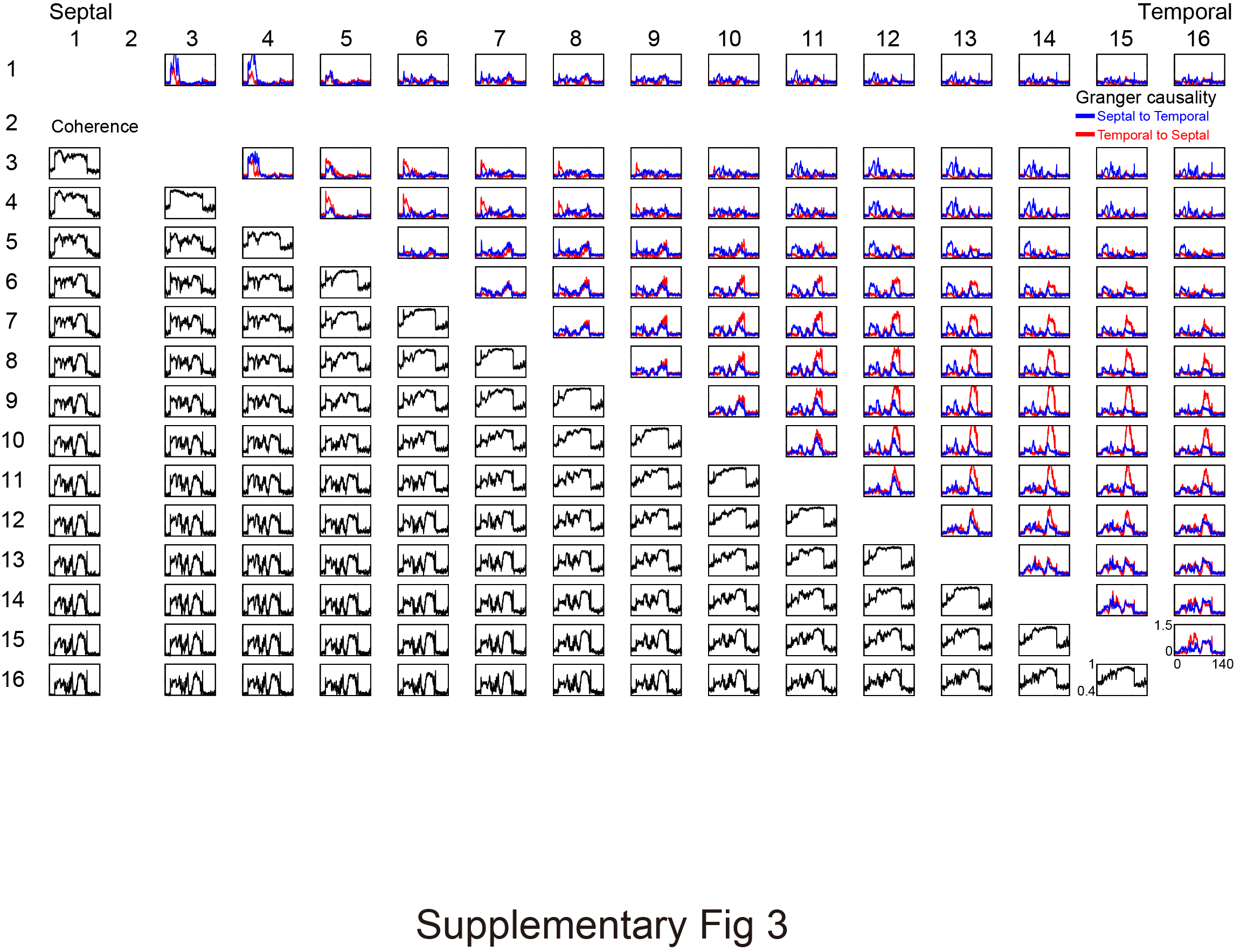

Supplement: Figure S3 — Examples of the Granger causality and coherence. Example traces of the Granger causality and coherence (rat 0624, trial 17). The numbers (1 to 16) at the left and upper part of the figure indicate the index of the recording sites on the probe. The site 1 is in the septal side of the hippocampus and nearest to the stimulation site. The site 16 is in the temporal side of the hippocampus and farthest to the stimulation site. The LFPs were recorded from these 16 recording sites (Recording site 2 is broken in this example experiment). The Granger causality and coherence were calculated in all LFP pairs. Right-upper panels are the Granger causality of the LFP pairs. The blue line indicates the causality from septal to temporal direction. The red line indicates the causality from temporal to septal direction. The left-lower panels are the coherence of the LFP pairs. The horizontal axis is time (second) for all panels. The photostimulation was applied from 10 to 40 seconds. Granger causality did not depend on the distance between electrode pairs, but rather on their relative position. Increase of the septal-to-temporal causality tended to occur in electrode pairs in the septal side, while increase of the temporal-to-septal causality did in the temporal side. Higher coherence was seen in closer pairs of electrodes. (TIF) [file pone.0060928.s003.tif]
